# Chronic Stress and Ovulatory Dysfunction: Implications in Times of COVID-19

**DOI:** 10.3389/fgwh.2022.866104

**Published:** 2022-05-23

**Authors:** Pilar Vigil, Jaime Meléndez, Hugo Soto, Grace Petkovic, Yanara A. Bernal, Santiago Molina

**Affiliations:** ^1^Reproductive Health Research Institute (RHRI), Santiago, Chile; ^2^Fundación Médica San Cristóbal, Santiago, Chile; ^3^Facultad de Medicina, Universidad San Sebastián, Santiago, Chile; ^4^University of Nothe Dame, Sydney, NSW, Australia; ^5^Facultad de Medicina Clínica Alemana, Universidad del Desarrollo, Santiago, Chile; ^6^Tallahassee Community College, Tallahassee, FL, United States

**Keywords:** menstrual cycle, kisspeptin, obesity, stress, anxiety, ovulation

## Abstract

Stress is known to be associated with adverse health outcomes. The COVID-19 pandemic and its associated lockdowns are examples of chronic stressors. Lockdown measures inadvertently caused significant psychological distress and became a powerful source of anxiety/stress, sleep disturbances, nutritional changes and weight gain. Stress is known to impact women's health specifically, through hypothalamic-pituitary-gonadal (HPG) axis dysfunction and resultant ovulatory dysfunction. Such dysfunction may manifest in menstrual irregularities and/or infertility due to hypothalamic hypogonadism. Here, we review the key physiological mediators of stress and associated ovulatory dysfunction. The kisspeptinergic system is comprised of sets of neurons located in the hypothalamus, the rostral periventricular region of the third ventricle (RP3V) and the arcuate nucleus (ARC). This system links nutrition, reproductive signals and stress. It plays a key role in the function of the HPG axis. During chronic stress, the kisspeptinergic system affects the HPG axis, GnRH pulsatility, and, therefore, ovulation. Leptin, insulin and corticotrophin-releasing hormone (CRH) are thought to be additional key modulators in the behavioral responses to chronic stress and may contribute to stress-related ovulatory dysfunction. This mini-review also summarizes and appraises the available evidence on the negative impact of chronic stress as a result of the COVID-19 pandemic lockdowns. It proposes physiological mechanisms to explain the observed effects on women's reproductive health and well-being. The review suggests areas for future research.

## Introduction

Ovulation is the result of the coordinated action of the endocrine, paracrine, and autocrine systems. Any disruption in the delicately coordinated interaction between the components of the hypothalamic-pituitary-ovarian axis may lead to ovulatory dysfunction (1). Persistent irregularities in the ovulatory cycle can be associated with stress, as well as with, endocrine, gynecological, autoimmune, nutritional, genetic, and iatrogenic disorders (2). Despite regular menses generally being considered an indicator of ovulation, they can, in fact, be associated with anovulation (3). Therefore, monitoring for regular ovulation, not just regular menstruation, is key when analyzing women's health.

Stress has many adverse health effects (4). Unfortunately, the relative lack of objective markers for chronic stress, means that identifying individuals suffering with chronic stress is very challenging clinically. In women, phenotypic markers of chronic stress include menstrual irregularities, amenorrhea, and/or infertility due to hypothalamic hypogonadism (5). Previous studies of the relationship between stress and menstrual cycle have yielded conflicting results. Some have found stress is associated with longer cycles, others with shorter cycles, and still others have found no association of stress and cycle length (6). It is interesting to note that the menstrual cycle changes observed with stress are sometimes similar to those experienced by women in the perimenopause.

The COVID-19 pandemic, and its lockdowns, have caused psychological distress, resulting in populations living under conditions of chronic stress (7–13). Lockdowns have been characterized by the development of negative lifestyles and their consequent metabolic changes (7). During the COVID-19 pandemic, women have been found to have a higher incidence of anxiety and depression disorders (14). This mini-review describes how the kisspeptinergic system integrates women's response to stress through its impact on energy balance and reproduction. Understanding such integration reveals how the stress associated with the COVID-19 pandemic may affect women's ovulatory cycles. This review evaluates the clinical evidence on this topic thus far and suggests areas for future research.

## Stress And Ovulatory Dysfunction

Ovulatory dysfunction is a group of disorders with variable clinical presentations that occasionally have serious long-term adverse effects. According to the World Health Organization (WHO), ovulation disorders are the main cause of infertility (82). These disorders fall into three categories: Group I ovulation disorders encompass hypothalamic insufficiency. Group II disorders involve HPO axis dysfunction and Group III constitutes ovarian insufficiency (15).

Group I ovulatory disorders include functional hypothalamic amenorrhea (FHA) (15–17). FHA is recognized as a sentinel indicator of chronic stress (4). FHA can also be triggered by excessive exercise or weight loss (16). In FHA, the final common pathway is activation of the limbic-hypothalamic-pituitary-adrenal axis (18) which then reduces the central gonadotropin-releasing hormone (GnRH) drive (19, 20). Stress and the resulting hormonal changes could trigger undernutrition or overnutrition, depending on fuel availability, attitudes toward food, and dietary behaviors such as binging, purging, overeating, or restricting. Reversal of functional hypothalamic amenorrhea includes restoration of ovulatory ovarian function and fertility (5).

Dysfunction of the HPO axis (Group II) constitutes 85% of ovulation disorders (15). Stress can trigger such dysfunction (4). It has been reported that prolonged or chronic stress in rats and human females can block, inhibit, or delay the preovulatory LH surge and thus disrupt the estrous or menstrual cycle (21). The endocrine system, including most specifically the hypothalamic-pituitary-adrenal (HPA) axis, and the immune system contribute to the development of these disorders (22–26).

## Physiology Of Stress

Stress primarily activates two systems: The sympathetic nervous system (SNS) and the Hypothalamic-Pituitary-Adrenal Axis (HPA). The activation of the SNS causes the release of Epinephrine and Norepinephrine. The activation of the HPA axis triggers a hormonal cascade in which corticotropin releasing hormone (CRH) is released by the hypothalamus, adrenocorticotropic releasing hormone (ACTH) by the anterior pituitary and finally, glucocorticoids by the adrenal gland. This results in an increase in the level of cortisol (27). Cortisol is released in order to increase glucose levels, which are needed to adequately respond to stressful situations. In order to achieve these levels of circulating glucose, cortisol promotes gluconeogenesis in the liver, the mobilization of amino acids from muscles and an increased lipolysis in the adipocytes (28). There is a strong inter-relationship between activation of the hypothalamo-pituitary-adrenal axis and energy homeostasis. Stress and glucocorticoids act to control both food intake and energy expenditure.

## Food Intake And Metabolic Signals

Under adequate nutritional conditions, the presence of metabolic signals such as insulin and leptin will activate anorexigenic neurons, as the POMC (pro-opiomelanocortin) neurons that release α-MSH (alpha-melanocyte stimulating hormone), causing satiety. On the other hand, insulin and leptin will inhibit the orexigenic neurons, which release NPY (neuropeptide Y) and AgRP (Agouti-related peptide), causing hunger (29). In overweight and obese individuals, high levels of leptin and insulin cause a state of resistance to both hormones, which, through positive feedback mechanisms, further increases their levels (30). Leptin and insulin resistance thus results in aberrant feedback signaling, causing the orexigenic neurons to release NPY and AgRP. This means the individual feels hungry, with the brain thinking more nutrition is needed, despite actually having excessive energy storage. These peptides are released into the arcuate nucleus of hypothalamus where reproductive signals are also sensed (31). The link between nutrition and reproductive signals is the kisspeptinergic system.

## The Kisspeptinergic System

**Figure 1:**
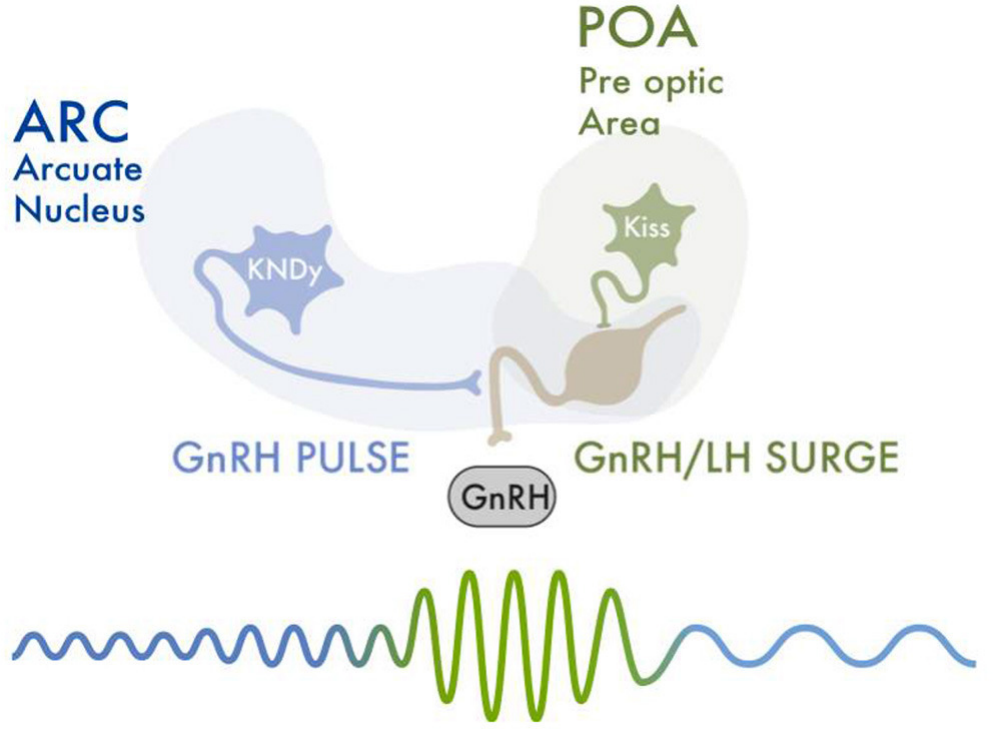
An increase in FSH levels leads to recruitment and development of ovarian follicles. Selected follicles produce rising estradiol levels. Estradiol and inhibin exert a negative feedback upon the HPG axis, thus decreasing FSH levels. Estradiol also causes a negative feedback upon the Kiss^ARC^ neurons. Kisspeptin-neurokinin B-dynorphin (KNDy) neurons present in the hypothalamus's arcuate nucleus (ARC), co-express neurokinin B (NKB) and dynorphin (Dy) and are essential for GnRH pulse generation and secretion. Low frequency hypothalamic GnRH pulses lead to a release of FSH and LH from the anterior hypophysis. One of the selected ovarian follicles becomes dominant, and secretes increasingly high levels of estradiol. This rapid and sustained increase in estradiol values gives the required signal to activate the Kiss^RP3V/POA^ neurons. This activation triggers the GnRH pulsatility and release, necessary for the LH/FSH surge. The LH surge is initiated, which causes follicular luteinization and an initial progesterone rise. Progesterone maintains the LH peak and is necessary for follicular rupture and adequate ovulation. After ovulation, estradiol levels abruptly decrease. This “turns off” the Kiss^RP3V/POA^ neurons, ending the LH/FSH surge.

The kisspeptinergic system consists of two populations of neurons in the hypothalamus; the rostral periventricular region of the third ventricle (RP3V) (also known as the preoptic area); and the arcuate nucleus (ARC) (also known as the infundibular nucleus). Both neuronal groups produce the neuropeptide kisspeptin, which plays a critical role in the function of the hypothalamic-pituitary-gonadal (HPG) axis (32). From these two areas, kisspeptinergic neurons release kisspeptin to GnRH neurons, which have kisspeptin receptors (Kiss1R). This stimulates GnRH neurons to release GnRH (33) (Figure 1).

Kisspeptinergic neurons located in the arcuate nucleus (Kiss^ARC^) are regulated mainly by metabolic inputs such as insulin, leptin and ghrelin (31). Kisspeptinergic neurons located in the anteroventral periventricular nucleus in the preoptic area (Kiss^RP3V/POA^) are regulated mainly by reproductive signals such as estradiol, testosterone and progesterone (34). Arcuate kisspeptin expression is similar in both sexes, whereas kisspeptinergic expression in the preoptic area is greater in females (35). When estradiol concentration is elevated, kisspeptin mRNA expression is increased in Kiss^RP3V^ neurons and decreased in the ARC nucleus (34). On the other hand, selective deletion of the classical progesterone receptor neurons in kisspeptinergic neurons prevents the LH surge. This suggests estrogens and progesterones act synergistically in kisspeptinergic neurons to modulate gonadotrophin release (36). The relationship between testosterone and the kisspeptinergic nuclei is not well established. In mammals, it has been shown that high levels of testosterone during prenatal development decrease the size of the preoptic kisspeptinergic nucleus area (34).

## Kisspeptinergic System As The Pacemaker Of The Menstrual Cycle

GnRH is released in a pulsatile pattern throughout all of the menstrual cycle, but the frequency and amplitude of its pulses differs with cycle phase. During the periovulatory period, there is an increase in the frequency and amplitude of GnRH pulses. Kisspeptinergic neurons induce such changes in the pattern of GnRH release (33). The increase in GnRH is generated by the activation of the Kiss^RP3V/POA^. These neurons respond to the increasing estradiol levels produced by the dominant follicle that occur around the periovulatory period (37). The concentration of estradiol that is produced by recruited follicles during the early follicular phase increases the pattern of secretion of kisspeptin by the Kiss^ARC^. Higher levels of estradiol are later produced by the dominant follicle and increase kisspeptin release by the Kiss^RP3V/POA^ (38). Such positive feedback of estradiol on kisspeptin release therefore increases the amplitude and frequency of GnRH production and secretion. This causes the LH surge. After ovulation, during the luteal phase, estradiol and progesterone modulate GnRH pulsatility by acting upon the Kiss^ARC^ (39).

## Stress And The Kisspeptinergic System

Under stress conditions, increased cortisol has an indirect inhibitory effect on Kiss^ARC^ neurons (40). This effect is mediated by the pro-opiomelanocortin and cocaine and amphetamine-regulated transcript (POMC/CART) neurons, located in the arcuate nucleus. These neurons, depending upon the stimuli they receive, secrete α-MSH. Alpha-MSH stimulates the Kiss^ARC^, or β-endorphins, which inhibit the Kiss^ARC^. Under stress conditions, these neurons detect CRH and cortisol. CRH and cortisol stimulate the production of β-endorphins over the production of α-MSH (16, 41). Beta-endorphins exert an inhibitory effect on Kiss^ARC^ neurons. Additionally, the deficit of α-MSH is sensed as an orexigenic stimulus (42). Another mechanism by which stress affects the kisspeptinergic system is through the increased expression and activity of *gonadotropin-inhibitory hormone*/*RFamide-related peptides* (GnIH/RFRP-3) (37, 43). GnIH/RFRP-3 is a peptide hormone that acts in the hypothalamus and pituitary gland. GnIH/RFRP-3 suppresses the synthesis and release of GnRH and gonadotropins (44). CRH, cortisol and GnIH inhibit the activity of Kiss ^ARC^ neurons, Kiss ^RP3V/POA^ neurons and GnRH neurons (45).

CRH is secreted by neurons in the anterior portion of the paraventricular nucleus of the hypothalamus. Under stress conditions, additional CRH-producing neurons are activated. This activation is greater in females than in males (46). This level of CRH expression in females has recently been associated with increased levels of anxiety (47). Data from non-human animal models also reveal extensive sex differences in CRH functions ranging from its presynaptic regulation to its postsynaptic efficacy (48). For example, females have greater CRH receptor turnover, post activation, than males. It has also been shown that at the locus coeruleus, which is involved in stress regulation and arousal, females have a greater number of neurons and dendritic projections (48–50). These differences make females more vulnerable to stress conditions (51). CRH is recognized as a key modulator of behavioral responses to stress. Therefore, sex differences in CRH processing may partly explain sex differences in responses to stress (48).

## Stress, Cortisol And Appetite-Related Hormones

Glucocorticoids released during stressful conditions act on the hypothalamus, increasing the sensation of hunger. This leads to an increase in appetite and food intake, especially intake of foods high in saturated fat and carbohydrates (52). When glucocorticoid levels remain high over time, such persistent eating changes can cause weight gain (53, 54). This mechanism is not fully elucidated. However, it has been suggested that during stress, the expression of POMC (an anorexigenic stimulus) is inhibited, and the expression of NPY and AgRP (orexigenic stimuli) is increased (55, 56). Individuals under chronic stress conditions also have higher levels of leptin, insulin, glucose and ghrelin (57).

**Figure 2:**
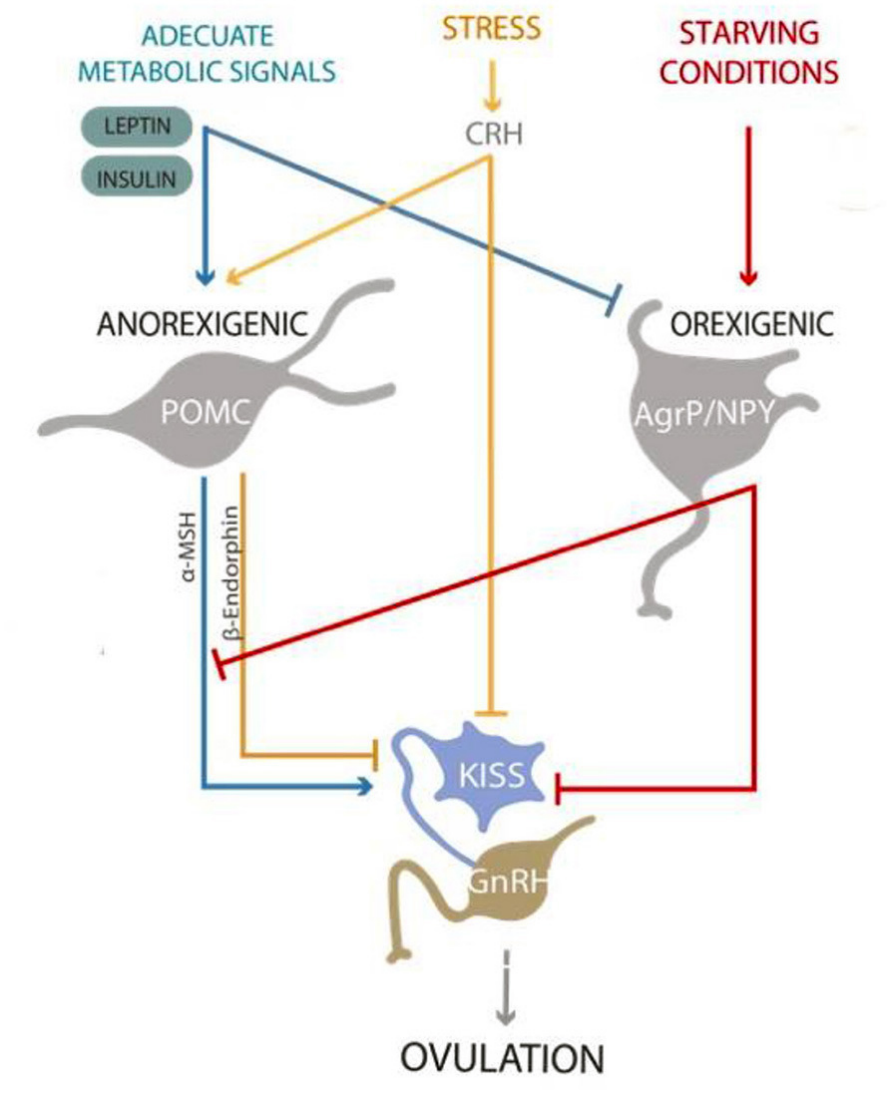
Kisspeptinergic neurons from the “preoptic area” and “arcuate nucleus” release kisspeptin to stimulate GnRH neurons to release GnRH. Under proper nutritional conditions, the presence of metabolic signals such as insulin and leptin will activate anorexigenic neurons, as the POMC neurons that release α-MSH, causing satiety. On the other hand, insulin and leptin will inhibit the orexigenic neurons, which release NPY and AgRP, causing hunger. Proper metabolic signals will stimulate the release of kisspeptin and promote ovulation. On the contrary, signs of starvation and/or stress will inhibit the release of kisspeptin, affecting ovulation and the reproductive process.

Sex is an important factor determining plasma leptin concentration. Women have markedly higher leptin concentrations than men (58). Elevated glucocorticoids, as found under stress conditions, affect leptin and insulin function and sensitivity. This contributes to the development of a leptin-insulin resistant state (59). Furthermore, in chronic stress situations, the release of LH and FSH is inhibited in both overweight and normal weight women. This contributes to ovulation inhibition (60, 61). An imbalance in leptin and insulin will also influence the kisspeptinergic system, thus affecting ovulatory function (Figure 2).

## Functional Hypothalamic Amenorrhea

Weight loss and weight gain, excessive physical exercise, and chronic stress induce an anovulatory state which is called “functional hypothalamic amenorrhea” (FHA). This condition is one of the main causes of secondary amenorrhea. It occurs when GnRH pulsatility is affected by a decreased activity of the Kiss^ARC^ neurons. This decreases the release of FSH and LH, generating a state of anovulation and hypoestrogenism (16, 61). The mechanisms underlying the pathophysiology of FHA are not fully understood. However, kisspeptin, NPY, ghrelin, leptin and, corticotropin-releasing hormone (CRH) are thought to play important roles in the physiological regulation of pulsatile GnRH secretion and thus are likely to be involved in the pathophysiology of FHA (62). As mentioned above, kisspeptin can directly stimulate GnRH secretion from the arcuate nucleus of the hypothalamus. The importance of the suppression of the kisspeptinergic system in FHA is further demonstrated by the fact that acute administration of kisspeptin to women with FHA potently stimulates gonadotropin release and ultimately restores ovulation (63).

## Stress And Lifestyle During Covid-19 Pandemic

The SARS-CoV-2 pandemic has affected millions of people worldwide. Many countries have adopted lockdowns or quarantines as strategies to help minimize the spread of the disease and the collapse of healthcare systems (64). The COVID-19 pandemic, and its lockdowns, have caused psychological distress, with populations living under conditions of chronic stress (7–13). It is important to note that chronic stress is a prolonged and constant feeling of stress that can negatively affect our health if it goes untreated and that state of emotional suffering associated with stressors and demands that are difficult to cope with in daily life lead to a psychological distress.

In the US, more than half university students reported moderate to severe anxiety symptoms during the pandemic (14). Severe anxiety symptoms were associated with increased hunger, emotional overeating and decreased enjoyment of food (14). Furthermore, obese people have reported, an excessive desire to eat during the pandemic (65). The lockdowns have caused so many changes in nutritional habits, sleep patterns, and physical activity routines that in the United States, people refer to the “Quarantine 15”. This phrase refers to the 15 pounds (6.8 kg) of weight that many Americans have gained during the lockdown (66). Individuals who reported changes in their eating behaviors during the pandemic also reported concurrent increases in depression (67). Stress is associated with an increase in high calorie food intake (68). This association is particularly strong in those who emotionally overeat and weaker or absent in those who have greater cognitive flexibility. Promoting cognitive flexibility and helping to prevent emotional overeating could help decrease the intake of high calorie foods during stressful conditions, such as the COVID-19 pandemic (68). Women are more vulnerable than men to developing anxiety (69–73). They are diagnosed twice as often as men and this prevalence increases with age and with the gradual decrease in estradiol secretion at menopause (74, 75). Therefore, tools to help prevent anxiety and pathological eating behaviors are like to particularly help women.

## The Lockdown And The Menstrual Cycle

There is a growing, though nascent, body of evidence appraising the impact of the COVID-19 pandemic and its impact on women's reproductive health. Whilst one cross-sectional study of 125 women did not find that the pandemic altered menstrual cycle characteristics, several other larger-scale studies have linked altered menses with stress associated with COVID-19 pandemic. The smallest of these studies included 263 participants with an average age of 26.3 ± 6.9 (18–45) (76). The authors found that menses duration and heaviness decreased in a statistically significant fashion during the COVID-19 pandemic. However, the clinical significance of such changes is unclear. Period time only decreased from 6.3 to 5.9 days and pads per day changed from 3.7 to 3.2. A larger cross-sectional study of 952 female healthcare workers in Turkey found that COVID-19 pandemic-induced anxiety, perceived stress, and depressive symptoms, were associated with increased menstrual cycle irregularity (77). Women with regular menstrual cycles for more than 1 year before the beginning of the pandemic were included in the study. During the COVID-19 pandemic, 71% of participants had regular menstrual cycles, and 23% had irregular menstrual cycles. This was a significant change given that all recruited women had regular menstrual cycles for at least 1 year. Covid stress scale scores (CSS) were significantly higher in women with irregular cycles than women with regular cycles. Depression, anxiety, and stress scores were likewise significantly higher in women with irregular cycles. This study was limited by the self-selecting nature of survey responders and by its reliance on women -recall of cycle characteristics. However, as the authors note, over 75% of their respondents used a period tracking application on a smart device which improves accuracy of women's self-reported data. A further observational study of over 1,000 women corroborated such findings (78). This study used a social media and text survey to appraise the effects of the COVID-19 pandemic on women. All study participants reported typical signs of chronic stress such as a significant increase in low mood, poor appetite, binge eating, poor concentration, anxiety, poor sleep, loneliness and excess alcohol use. Women also reported a median 2 kg increase in self-reported body weight. Forty-six percent of participants reported a change in their menstrual cycle since the beginning of the pandemic and 53% described a worsening of premenstrual symptoms. Indeed, one third of participants reported new dysmenorrhea during the pandemic. Interestingly, whilst median cycle length and days of bleeding were unchanged, total cycle variability was increased.

The largest study of over 18,000 mobile app users similarly found that stress was reported in nearly half of participants during the COVID-19 pandemic (79). Interestingly, whilst a number of participants recorded more anovulatory cycles (7.7%) or cycles of abnormal length (19.5%) during the pandemic, a number of women actually recorded fewer anovulatory (9.6%) or abnormal length cycles (19.6%). The authors suggest that this may reflect that the COVID-19 pandemic likely affected women with different sociodemographic characteristics differently. For example, app-users in the study were typically from high-income countries (USA and Great Britain) and had high education levels. Therefore, a number of these women may have started working from home, rather than commuting. Studies have shown that women who began working from home, rather than commuting, may have had an increased opportunity to exercise or eat healthily, given the reduced commute time (80). Overall, these studies suggest there is an association between the COVID-19 pandemic-induced anxiety and increased prevalence of menstrual cycle irregularities in women. However, they also highlight that the COVID-19 pandemic measures did not affect all women equally.

## Future Directions And Perspectives

The evidence evaluating the impact of the COVID-19 pandemic on women's reproductive health is still nascent and the consequences for women's reproductive health are just emerging. The medium- and longer-term effects of the pandemic remain to be seen. Even as lockdown-related stress recedes, chronic stress as a result of other factors (e.g., financial stressors) related to the pandemic may remain. The studies appraised in this mini-review were largely conducted at the beginning of the COVID-19 pandemic. It will be important to consider how stress levels develop and change at later stages of the pandemic and during post-pandemic recovery, as well as how such stress levels may influence women's menstrual cycles, reproductive health and well-being.

The studies were also limited in a number of other ways. Firstly, all the studies are based on self-reported personal data. Such self-reporting may have inaccuracies. In particular, pre-pandemic menstrual markers were often collected retrospectively and so were subject to recall bias. This also prevented rigorous controls for any changes in women's socio-economic and educational levels pre vs. during pandemic. Furthermore, a number of studies did not consider the effect of SARS-CoV-2 exposure or infection. This could present a confounding physiological factor. This is likely particularly important for studies that focused on female healthcare workers (77). Furthermore, many women in some studies were from a high socioeconomic group. This limits the general applicability of such work. For example, one study found that women with high levels of education (e.g., a PhD degree) had higher levels of increased perceived stress (48.9%) compared with women with a high school degree or lower (40.3%) (79).

The most significant limitation of these studies however was the lack of clinical laboratory data to determine objective measures of e.g., stress hormones and their impact on ovulation. For example, perceived stress may differ from physiologically high levels of stress. Clarifying whether women who feel more stressed show higher levels of stress hormones would be interesting. Furthermore, it is known that in chronic stress, the acute stress response may actually be blunted (81). Might this result in counter-intuitive impacts on women's ovulatory cycles?

Only one study used an additional biomarker (basal body temperature) in an attempt to record anovulatory cycles directly (79). Other papers focused on menses length and length of menstrual cycle. Total menstrual cycle length could be unchanged, but luteal and/or follicular phase, specifically might be impacted. As discussed in the introduction, ovulatory dysfunction can occur even when menstruation remains regular. More studies that track ovulatory function directly, through progesterone measurements, cervical fluid recordings or basal body temperature are needed. These studies could offer greater clarity and insight into women's health during times of high stress.

One strength of these studies was that the majority of papers did exclude women using hormonal contraceptives (76, 77, 79). This would have made it easier to observe physiological responses to stress on the menstrual cycle. It would be interesting to observe how hormonal contraception might impact perceived stress and physiological response.

Other areas for further research could involve whether times of chronic stress impact all women equally. For example, Sadler's work suggests that women who emotionally overeat may respond differently to stress (68). It would be interesting to further investigate this link and to elucidate whether its origins are genetic or social or both. Such work could allow women who are particularly vulnerable to stress to be identified and helped earlier. Finally, future work could look at how to best manage ovulatory dysfunction associated with chronic stress. For example, how might therapies target stress perception and management (e.g., cognitive behavioral therapy) or physiological markers of ovulatory dysfunction (e.g., hypoestrogenism in the case of FHA)?

## Conclusion

During this time, a number of women have exhibited changes in their menstrual cycles. Many women have reported a worsening of premenstrual symptoms. This highlights the link between mental status and the reproductive axis. Monitoring their cycles more closely may allow women to identify alterations in their hormonal balance which might confirm or even indicate their stress levels. This mini-review has presented evidence that the COVID-19 pandemic has negatively affected women's reproductive health through the possible ovulatory dysfunction. Further work should focus on using biomarkers to better evaluate the nature of such dysfunction. Discerning which women are likely to be most at risk and benefit most from targeted therapies (e.g., cognitive behavioral therapy) may offer great help in the future. Even as the pandemic recedes, it is important to remember that women experience periods of acute and chronic stress across the world due to other factors. These can include war, famine and displacement. We hope that findings during the COVID-19 pandemic may allow us to give a better healthcare to women in the future.

## Author Contributions

PV and JM: writing, review, and revision of the manuscript. HS and YB: bibliographic search and writing. SM: review and revision of the manuscript. All authors contributed to the article and approved the submitted version.

## Conflict of Interest

The authors declare that the research was conducted in the absence of any commercial or financial relationships that could be construed as a potential conflict of interest.

## Publisher's Note

All claims expressed in this article are solely those of the authors and do not necessarily represent those of their affiliated organizations, or those of the publisher, the editors and the reviewers. Any product that may be evaluated in this article, or claim that may be made by its manufacturer, is not guaranteed or endorsed by the publisher.
